# The gut-lung axis in critical illness: microbiome composition as a predictor of mortality at day 28 in mechanically ventilated patients

**DOI:** 10.1186/s12866-023-03078-3

**Published:** 2023-12-18

**Authors:** Piaopiao Zhou, Zhiqiang Zou, Wenwei Wu, Hui Zhang, Shuling Wang, Xiaoyan Tu, Weibin Huang, Cunrong Chen, Shuaijun Zhu, Qinyong Weng, Shixiang Zheng

**Affiliations:** Department of Critical Care Medicine, Fujian Medical University Union Hospital, Fuzhou, China

**Keywords:** Intestinal microbiota, Lung microbiota, Gut-lung axis, Intensive care unit, Longitudinal study

## Abstract

**Background:**

Microbial communities are of critical importance in the human host. The lung and gut microbial communities represent the most essential microbiota within the human body, collectively referred to as the gut-lung axis. However, the differentiation between these communities and their influence on clinical outcomes in critically ill patients remains uncertain.

**Methods:**

An observational cohort study was obtained in the intensive care unit (ICU) of an affiliated university hospital. Sequential samples were procured from two distinct anatomical sites, namely the respiratory and intestinal tracts, at two precisely defined time intervals: within 48 h and on day 7 following intubation. Subsequently, these samples underwent a comprehensive analysis to characterize microbial communities using 16S ribosomal RNA (rRNA) gene sequencing and to quantify concentrations of fecal short-chain fatty acids (SCFAs). The primary predictors in this investigation included lung and gut microbial diversity, along with indicator species. The primary outcome of interest was the survival status at 28 days following mechanical ventilation.

**Results:**

Sixty-two mechanically ventilated critically ill patients were included in this study. Compared to the survivors, the diversity of microorganisms was significantly lower in the deceased, with a significant contribution from the gut-originated fraction of lung microorganisms. Lower concentrations of fecal SCFAs were detected in the deceased. Multivariate Cox regression analysis revealed that not only lung microbial diversity but also the abundance of *Enterococcaceae* from the gut were correlated with day 28 mortality.

**Conclusion:**

Critically ill patients exhibited lung and gut microbial dysbiosis after mechanical ventilation, as evidenced by a significant decrease in lung microbial diversity and the proliferation of *Enterococcaceae* in the gut. Levels of fecal SCFAs in the deceased served as a marker of imbalance between commensal and pathogenic flora in the gut. These findings emphasize the clinical significance of microbial profiling in predicting the prognosis of ICU patients.

## Introduction

The healthy individuals, the microbiota displays impressive stability and distinctiveness among various anatomical regions, encompassing the gut, lungs, nasal cavity, and vagina [1–3]. The microorganisms residing in the lungs and gut serve vital functions in preserving the host's structural integrity, regulating nutrient metabolism, and defending against bacterial pathogens [4–7].

The gut and lung microbiota are interconnected through the gut-lung axis [8–11]. The gut microbiota regulates immune responses in mucous membranes and distant organs, including the lungs [12, 13]. Metabolites such as SCFAs, the neuroendocrine system, and various pathways are involved in the modulation of the immune and inflammatory responses of the body by the gut microbiota [14–16]. Currently, microbiological research is focused on specific strains of gut commensal microorganisms known as "probiotics", which are thought to positively influence host immunity and/or fight pathogenic microorganisms [17–19]. Recent studies have shown that reduced production of SCFAs by gut commensal bacteria stimulates lung inflammation [20]. However, this interaction in critically ill patients is still limitedly recognized [21].

Critically ill patients require a variety of treatments in the ICU, including antibiotic therapy, mechanical ventilation, continuous blood purification, immunosuppressive therapy and other non-invasive interventions [22]. Therefore, the microbial composition and diversity of patients are affected [23–25]. Microbial dysbiosis may affect disease progression, drug response, systemic inflammatory response and organ dysfunction in critically ill patients [26–29]. The lungs and gut contain the largest and most important microbial communities in the human body, impacting human health, especially disease severity and mortality [30–33]. A deep understanding of lung and gut microbial interactions is essential for understanding the underlying mechanisms and implementing preventive measures to improve disease prognosis [34, 35].

Microbiological studies in the field of critical care are still relatively rare, mainly due to the complex clinical characteristics of critically ill patients, who exhibit a high degree of heterogeneity in microorganisms [36]. In addition, most studies only analyze microbiome samples from a single anatomical site or non-chronologically sequential samples, which limits the understanding of microbial dynamics [37–39]. To gain a comprehensive understanding of microbial characterization in the gut-lung axis in critically ill patients, we conducted an observational study analyzing microbes from two anatomical sites (lungs and gut) at two time points (within 48 h and 1 week after mechanical ventilation) and examining metabolites of gut microbes, which are SCFAs. we aimed to characterize the dynamics of changes and interactions between the lung and gut microbiomes and to relate its association with patient mortality at 28 days.

## Methods and materials

### Patient enrollment

We conducted an observational study in the ICU of the Fujian Medical University Union Hospital, which admits medical, surgical, neurosurgical, and trauma patients and treats approximately 1,000 patients per year.

**Fig. 1:**
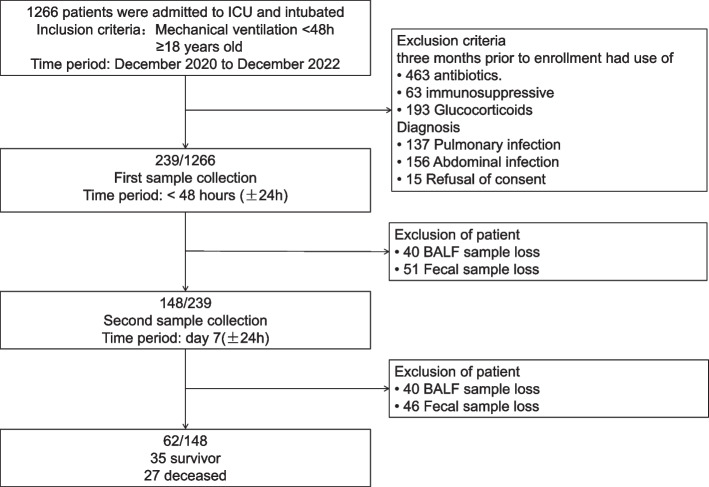
Process of subject enrollment

From December 2020 to December 2022, we recruited adult patients without pulmonary and abdominal infections who were receiving invasive mechanical ventilation, who had been intubated for less than 48 h, and who were expected to require mechanical ventilation for more than 4 days. We excluded patients who were pregnant, had received antibiotics, immunosuppressants, or glucocorticoids in the past 3 months through enrollment, refused to participate, or lacked adequate clinical data. Patients with poor or insufficient samples due to delayed fiberoptic bronchoscopy, constipation, etc. were considered as sample loss and were excluded. Two pairs of bronchoalveolar lavage fluid (BALF) and stool samples were requested from patients during their stay in the intensive care unit, and sample collection was automatically stopped once the patient was discharged from the hospital or no longer required mechanical ventilation. If samples were not available within the specified time, samples from within 24 h (still within 48 h ± 24 h of mechanical ventilation and Day 7 ± 24 h) were used. The detailed procedure is shown in Fig. 1.

### Data collection

Age, gender, basic clinical information, antimicrobials and routine BALF culture, catecholamine dose, and a score of organ failure (SOFA) were extracted from the medical records. All patients were deeply sedated at the beginning of intubation with a GCS score of < 6 and a neurological score of 4 on the SOFA scale. Ventilator parameters were represented as PEEP levels. Acute respiratory distress syndrome (ARDS) was defined according to the Berlin criteria and evaluated by two attending physicians. Clinical outcomes, including 28-day mortality, duration of mechanical ventilation, and length of stay in the ICU, were also recorded.

### Sample collection

Lower respiratory tract samples can be used to study the lung microbiota of individuals [40, 41]. For clinical diagnostic and therapeutic purposes, approximately 10–20 ml of BALF were obtained by fiberoptic bronchoscopy. All operations were performed by an experienced physician and obtained according to protocols. The first tube was discarded and the second tube was divided into two and labeled with information and code on the bottle; one was sent to our laboratory for conventional culture and the other was immediately stored in a -80 °C refrigerator for microbial 16sRNA sequence detection.

After washing hands and wearing gloves, use the sampling spoon of the fecal collection tube to take the inside of the middle portion of the stool, approximately 0.5–10 g (the surface of the stool contains detached intestinal mucosal cells). The bottles were labeled and coded. All samples were stored in a -80 °C refrigerator immediately after collection for microbiological testing.

### DNA extraction, bioinformatics analysis and taxonomy annotation

We isolated bacterial DNA directly from BALF and fecal samples, targeting the V3 + V4 region of the ribosomal RNA gene using PCR [42]. Microbial DNA was extracted using the HiPure Soil DNA Kits (or HiPure Stool DNA Kits) (Magen, Guangzhou, China) according to the manufacturer’s protocols. The resulting amplicons were purified and pooled in equimolar before being subjected to paired-end sequencing (PE250) on an Illumina platform according to standard protocols. The raw reads were deposited into the NCBI Sequence Read Archive (SRA) database. Adapters and low-quality reads were removed as they can affect downstream analyses, leaving us with a set of high-quality clean tags [43]. These tags were then clustered into operational taxonomic units (OTUs) with at least 97% similarity using the UPARSE (version 9.2.64) pipeline [44]. We removed any chimeric tags using the UCHIME software and finally obtained a set of effective tags for further analysis. To represent each cluster, the tag sequence with the highest abundance was selected.

### Microbial analysis and statistical analysis

After processing the sequencing data, we utilized the vegan software package version 2.5.3 to analyze microbial ecology. The abundance statistics for each taxonomic group were visualized using Krona version 2.6 [45], and the stacked bar plot of community composition was generated using the gplot2 package version 2.2.1 in the R project [46]. The Shannon index was computed using QIIME version 1.9.1. Furthermore, we conducted a Venn diagram analysis using the vegan package in R, considering a species present in a group only when the mean tag count of the OTU/species was greater than 1 in that group. To generate the species abundance heatmap, we employed the Pheatmap package in R, which displays species with a relative abundance of at least 0.1% in at least one sample.

For beta diversity analysis, the distance between samples was calculated using Bray–Curtis. Beta diversity was analyzed using principal coordinate analysis (PCoA) and non-metric multidimensional scaling analysis [47]. To detect statistical differences in Beta diversity metrics between groups, we computed permutation multivariate analysis of variance (PERMANOVA) and analysis of similarity (ANOSIM) using the Vegan package version 2.5.3 in the R project [48]. We performed between-group species comparisons using Welch's t-test and Wilcoxon rank sum test in the Vegan package version 2.5.3, as well as Kruskal–Wallis H test in the same package. We performed differential abundance analyses at the family level using Analysis of Compositions of Microbiomes with Bias Correction (ANCOM-BC). SourceTracker 1.0.1 with QIIME was used to assess the contribution of gut microbiota to the lung microbiota. A *P* value of < 0.05 was considered statistically significant.

Quantitative variables are presented as medians and interquartile ranges (IQR) and were compared with the use of the Mann–Whitney Wilcoxon rank-sum test. Continuous parameter data were expressed as mean values (standard deviations, SDs) and analyzed using Student t or One-way analysis of Variance (ANOVA) tests. Categorical variables were expressed as the number of patients (percentages) and were compared with the use of the chi-square test or Fisher's test. Univariate and multifactorial Cox proportional risk models were used to analyze the risk factors for 28-day mortality.

### Bioinformatics analysis methods of SCFAs and statistical analysis

We measured the levels of SCFAs in stool samples within 48 h of mechanical ventilation using Waters Acquity UPLC and AB SCIEX 5500 QQQ-MS. We used multivariate statistical analysis OPLS-DA VIP values and univariate statistical analysis T-test *P* values to identify significant differences. Those with a *p* value of T test < 0.05 and VIP ≥ 1 were considered differential lipids between the two groups.

## Result

The study enrolled 62 patients, of whom 27 died (44%) and 35 survived (56%) after 28 days. Each patient provided two sets of BALF and stool samples, one within 48 h (intubation group) and the other at one week after mechanical ventilation (Day 7 group). Table 1 shows the clinical characteristics of the patients. The PEEP of ventilator parameters was higher in the deceased compared to the survivors. However, there were no significant differences between the two groups in terms of clinical characteristics, culture results or antibiotic use.

**Table 1:** Baseline characteristics and outcome

	Deceased(27)	Survival(35)	P
Age	63(53, 69)	58(45, 75)	0.121
length of ICU stay(d)	17(11, 23)	13(8, 23)	0.286
level of PEEP	8.78 ± 3.85	6.54 ± 2.09	0.005
Days of mechanical ventilation	16.54 ± 8.75	13.93 ± 7.08	0.210
Enteral nutrition time(d)	2(1, 4)	2(1, 4)	0.896
SOFA	14(12, 16)	13(8, 14)	0.098
Antifungal	11	11	0.447
Sex(Male)	15	17	0.585
Emergency Surgery	11	12	0.602
Smoking history	7	6	0.400
Alcohol history	7	7	0.580
VAP	17	19	0.492
ARDS	21	22	0.206
Early enteral nutrition (72 h)	20	26	0.985
Diagnosis-abdominal surgery	3	8	0.321
Diagnosis-hematopathy	3	4	1.000
Diagnosis-Rheumatic immune disease	2	3	1.000
Diagnosis-respiratory disease	9	5	0.124
Diagnosis-neurosurgery	10	10	0.480
Diagnosis-respiratory disease	0	5	0.063
complication-hypertension	4	5	1.000
complication-diabetes	4	3	0.698
complication-cardiovascular disease	5	4	0.485
complication-tumour	4	5	1.000
complication-Neuropathy	4	6	1.000
Catecholamine			0.371
Not in Use	4(38.5%)	9(69.2%)	
Dopamine ≤ 5ug/kg/mim	8(61.5%)	5(38.5%)	
Dopamine > 5ug/kg/mim or adrenal dose ≤ 0.1ug/kg/min or norepinephrine dose ≤ 0.1ug/kg/min	4(33.3%)	8(66.7%)	
Dopamine > 15ug/kg/mim or adrenal dose > 0.1ug/kg/min or norepinephrine dose > 0.1ug/kg/min	11(45.8%)	13(54.2%)	
Antibiotics			
Carbapenems	16	16	0.290
Cephalosporins	9	13	0.756
β- Lactam antibiotics	12	14	0.725
Tegacyclin	9	6	0.140
Aminoglycoside	0	2	0.500
Anti positive bacteria	16	16	0.290
Quinolone	3	3	1.000
Polymyxin	2	3	1.000
Anti anaerobic bacteria	1	0	0.436
BALF traditional culture			
Enterobacter aerogenes	6	6	0.616
CRAB	3	7	0.491
CRKP	14	18	0.974
CRPA	6	7	0.831
MRSA	4	10	0.199
Neisseria	5	5	0.735
Stenotrophomonas maltophilia	4	4	0.719
Burkholderia cepacia	2	3	1.000
Chryseobacterium indologenes	3	3	1.000

Data presented as median with [IQR]

*PEEP* positive end-expiratory pressure, *SOFA* Sequential Organ Failure Assessment. Anti-positive bacteria: vancomycin or linezolid. Anti anaerobic bacteria: ornidazole. *VAP* Ventilator associated pneumonia, *ARDS* acute respiratory distress syndrome, *CRAB* Cabapemne Resistant Acinetobacter Baumannii, *CRKP* carbapenem-resistant Klebsiella pneumoniae, *CRPA* Carbapenem-resistant Pseudomonas aeruginosa, *MASA* methicillin-susceptible Staphylococcus aureus

### Comparison of microbial alpha diversity in survivors and deceased

**Fig. 2:**
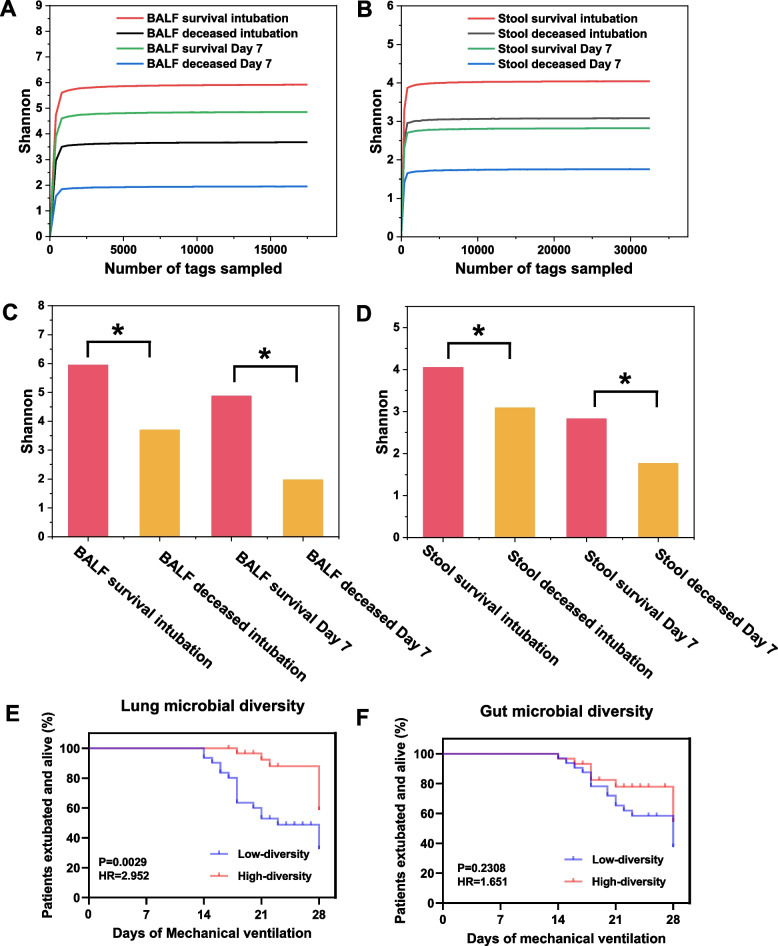
Dilution curves for lung (**A**) and gut (**B**) microbiota are shown. Flat curves indicate that the sequencing depth was sufficient and that increasing sequencing depth no longer had a significant effect on species diversity; The Shannon Index was used to assess the microbial diversity of BALF (**C**) and fecal microbiota (**D**) in the survival (red) and deceased (orange) patients in the intubated and day 7 groups; Kaplan–Meier curves were used to estimate the association of microbial diversity with extubation and survival within 28 days. Patients were stratified according to the alpha diversity (median subgroup of the Shannon index) of their lung (**E**) and gut (**F**) microbiota at the time of intubation. *p < 0.05

A total of 247 samples, including 123 BALF and 124 fecal samples, were subjected to 16S rRNA gene sequencing. BALF **(**Fig. 2A) and feces (Fig. 2B) were diluted to saturation levels, indicating sufficient sample coverage and distribution. The α-diversity estimated with the Shannon index reflects the diversity and abundance of the microbiota. The α-diversity in the BALF and fecal microbiota showed a decreasing trend over time, with the deceased group having significantly lower microbial diversity in both the lungs (Fig. 2C) and the gut (Fig. 2D) compared with the survival group (*P* < 0.05). To further investigate the relationship between α-diversity and clinical outcomes, we analyzed the Kaplan–Meier curves of microorganisms in the lung (Fig. 2E) and gut (Fig. 2F) at the time of intubation. The results showed that mortality was higher in patients with low α-diversity of lung microorganisms(*P* < 0.01).

### Comparison of the relative abundance of BALF and fecal microbiota in survivors and deceased

**Fig. 3:**
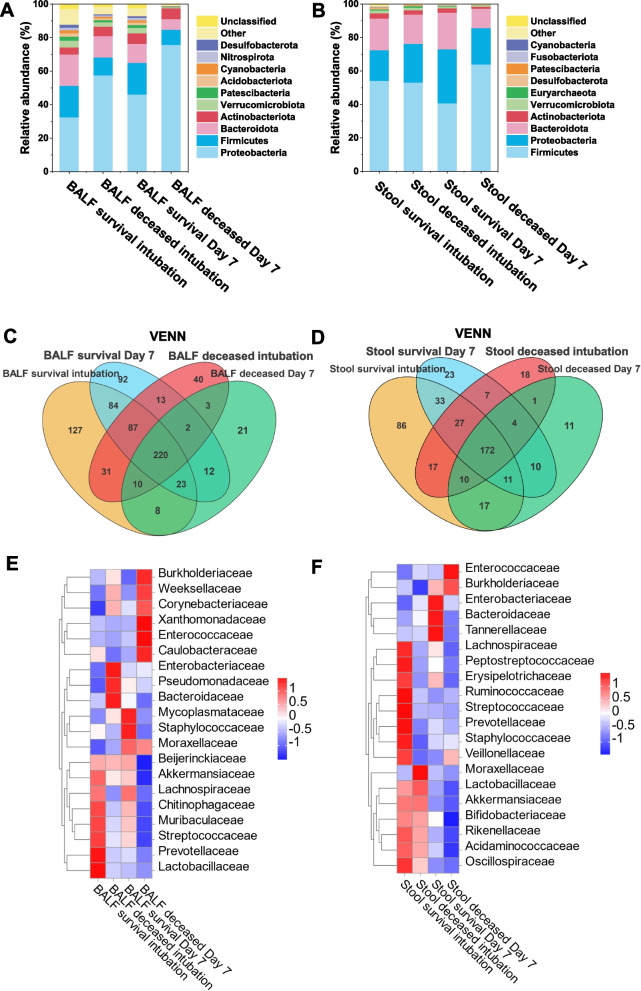

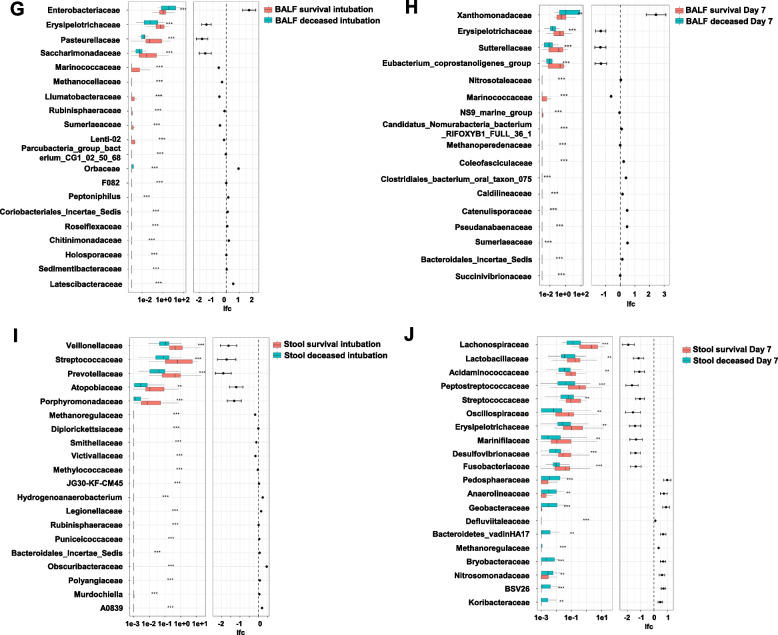
Stack maps of species distributions representing the relative abundance of the top 10 taxa of BALF (**A**) and fecal (**B**) microorganisms, expressed at the phylum level; Venn diagrams of the lung (**C**) and gut (**D**) microbiota of the two groups are given, showing shared genera between survivor and deceased populations in overlapping regions, and unique genera in non-overlapping regions; Heatmap of hierarchical clustering of lung (**E**) and gut (**F**) microbiota, with each row representing a species and each column representing survivors and deceased in both groups. The color indicates the abundance of the species, with dark blue indicating lower abundance and red indicating higher abundance; Paired differential abundance analysis of lung (**G**, **H**) and gut (**I**, **J**) microbes stratified by survivors and deceased. Natural log transformed sampling-bias corrected observed abundances, as used in ANCOM-BC, were used here to generate the boxplots. The boxplots show the distribution of data for survivors (red) and deceased (blue). Each sample is plotted based on the abundance of its species. The left, center, and right vertical boundaries of each box represent the first, second (median), and third quartiles of the data. W: test statistics. W = lfc/se. p_val: *p*-values. *P*-values are obtained from a two-sided Z-test using the test statistic W. q_val: adjusted *p*-values. Adjusted *p*-values are obtained by applying p_adj_method to p_val. lfc: log fold changes obtained from the ANCOM-BC log-linear (natural log) model. **p < 0.01,***p < 0.001

The microbial composition of the Lung and Gut was analyzed to identify the major microbial taxa at each site. In the lung, Proteobacteria, Firmicutes, *Bacteroidota*, and *Actinobacteriota* emerged as the dominant phylum, together accounting for nearly 70% of the total sequenced reads. the gut samples *Firmicutes*, *Proteobacteria*, and *Bacteroidota* dominated, accounting for nearly 90% of the total sequenced reads. Compared to the survivors, BALF samples from the deceased had higher abundance of *Proteobacteria* (intubation group:32.5654% vs. 57.5656%; Day 7 group:46.0896% vs. 75.8838%), while the opposite was true for the *Firmicutes* (intubation group:18.8191% vs. 10.8256%; Day 7 group:19.0444% vs. 9.0092%) (Fig. 3A). In fecal samples, the difference was not significant in the intubation group. However, when on Day 7 group, compared to the survival, there was an increase in *Firmicutes* in the deceased (40.8465% vs. 64.0591%), and there was a decrease in both the *Bacteroidota* (21.9145% vs. 11.5909%) and *Proteobacteria* (32.2428% vs. 21.6599%) (Fig. 3B). The Venn diagram reveals a substantial reduction in specific bacterial genera in the deceased group compared to the surviving group, except shared microbes (Fig. 3C, D). Heat maps showed that the lung and gut of the survival were dominated by common commensal bacteria, while the deceased were characterized by an enrichment of intestinal flora such as *Enterobacteriaceae* and *Enterococcaceae* (Fig. 3E, F).

The microorganisms identified using ANCOM-BC that were most enriched when intubated in the lungs of survivors were *Erysipelotrichaceae* (W = -4.1244, *p* < 0.0001, q < 0.01), *Pasteurellaceae* (W = -4.2165, *p* < 0.0001, q < 0.01) and *Saccharimonadaceae* (W = -3.4762, *p* < 0.001, q < 0.05) (Fig. 3G). At Day 7 Sutterellaceae (W = -3.6211, *p* < 0.001, q < 0.05), *Eubacterium_coprostanoligenes_group* (W = -3.3317, *p* < 0.001, q < 0.05) and *Marinococcaceae* (W = -1.5509, *p* < 0.001, q < 0.05) were enriched (Fig. 3H). Survivors had a predominance of intestinal commensal bacteria and were enriched for *Prevotellaceae* (W = -4.4212, *p* < 0.0001, q < 0.01), *Veillonellaceae* (W = -3.6317, *p* < 0.001, q < 0.05), *Porphyromonadaceae* (W = -3.4590, *p* < 0.001, q < 0.05), *Streptococcaceae* (W = -3.4473, *p* < 0.001, q < 0.05) (Fig. 3I). At Day7 enrichment of *Lachnospiraceae* (W = -4.2206, *p* < 0.0001, q < 0.01), *Peptostreptococcaceae* (W = -3.5600, *p* < 0.001, q < 0.01), Lactobacillaceae (W = -2.9182, *p* < 0.01, q < 0.05), *Acidaminococcaceae* (W = -2.8094, *p* < 0.01, q < 0.05) (Fig. 3J).

### Comparison of microbial beta diversity in survivors and deceased

**Fig. 4:**
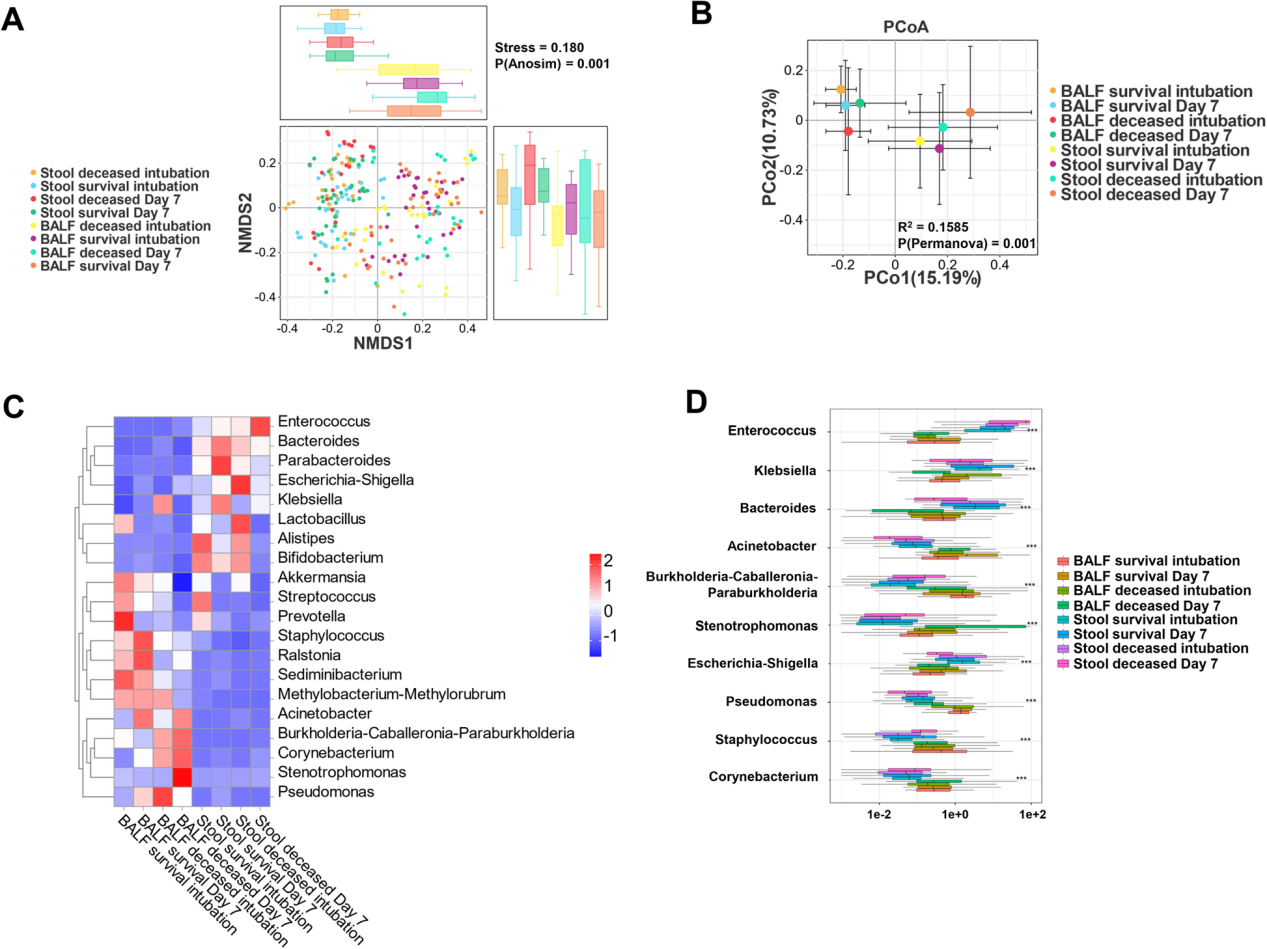
The non-metric multidimensional scaling (NMDS) plot (**A**) and principal coordinate analysis (PCoA) model (**B**) demonstrate the group differences of the Bray–Curtis distance index between the survivor and deceased groups, which are presented in the form of table/box plots. Each row and column represents a sample, and the color of each square indicates the corresponding distance between samples; The hierarchical clustering heatmap of shared lung and gut microbiota at two different time points is presented(**C**); Differential abundance analysis of lung and gut genus levels by survival and deceased. Natural log transformed sampling-bias corrected observed abundances, as used in ANCOM-BC, were used here to generate the boxplots (**D**), ***p < 0.001

Beta diversity is a measure of species diversity among ecosystems, according to the absence or presence of species and their abundance. To further clarify the association between respiratory and intestinal microorganisms, we used NMDS (Fig. 4A) and PCoA (Fig. 4B) to construct a model for differential analysis of specimen clustering results. It is known that biogeographic barriers exist between the respiratory tract and intestine microbial communities within individuals. There were significant differences between lung and gut microbes (*p* < 0.01). In the survival group, the commensal bacteria Akkermansia, Streptococcus, *Prevotella*, and *Lactobacillus* were enriched in both the lungs and gut, whereas an increase in pathogenic bacteria and a decrease in commensal bacteria predominated in the deceased (Fig. 4C). The deceased group was enriched with the pathogenic *Enterobacteriaceae* commonly found in reality: *Klebsiella* (w = 59.3149, *p* < 0.0001, q < 0.01), *Escherichia-Shigella* (w = 65.3208, *p* < 0.0001, q < 0.01). A significant increase in *Enterococcus* (w = 318.1675, *p* < 0.0001, q < 0.01) was noted in the feces of the deceased (Fig. 4D).

### Comparison of lung microbiology from gut microbial sources in survivors and deceased

**Fig. 5:**
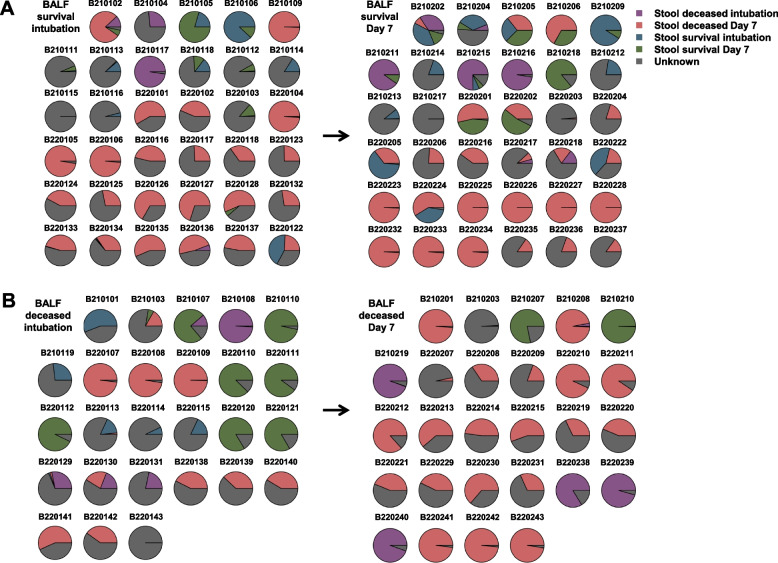
Percentage tributes of bacterial sources in lung samples from survivors and deceased. SourceTracker results are shown as pie charts for each patient sample. Sources: Stool deceased intubation, Stool deceased Day 7, Stool survival intubation, Stool survival Day 7 and unknown sources (Unknown)

SourceTracker was used to analyze all patient microbiome samples for community source proportions. The source samples came from the patient's lungs at the time of intubation and on DAY 7. First, fecal microbes contributed significantly to BALF microbes. In both surviving and deceased patients, nearly 2/5 of BALF microbes had more than 50% fecal origin during the intubation period, but the percentage continued to increase with longer intubation time, accounting for approximately 3/5 of BALF. Second, in the surviving group, the fecal origin of microbes in the BALF was much more varied, with the majority being unknown and surviving group fecal microbes (Fig. 5A). The BALF microorganisms in the deceased group on Day 7 were dominated by their contemporaneous feces, presumably the lung and gut microorganisms in the deceased were more similar (Fig. 5B).

### Comparing the fecal SCFAs concentrations in survivors and deceased

**Fig. 6:**
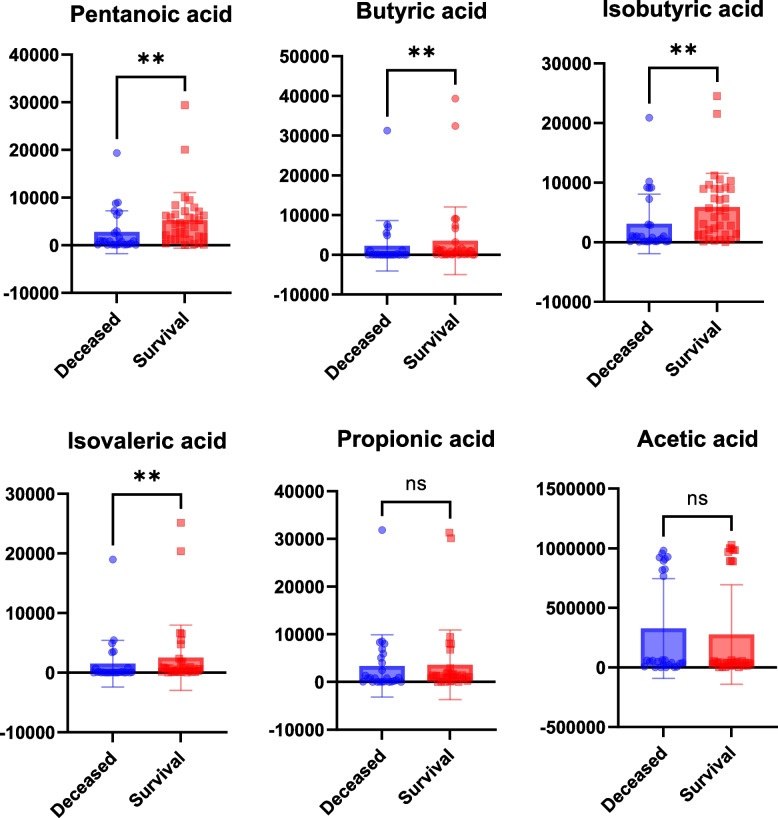
Scatter box whisker plots of SCFAs concentrations in survivors and deceased, **p < 0.01

SCFAs are metabolites of dietary fiber fermentation by commensal gut bacteria and have anti-inflammatory, immunomodulatory and distal lung protective effects. We quantified seven SCFAs in the feces of the intubation group, including Caproic acid, Pentanoic acid, Butyric acid, Isobutyric acid, Isovaleric acid, Propionic acid and Acetic acid. Concentrations of Pentanoic acid, Butyric acid, Isobutyric acid, and Isovaleric acid were significantly lower in the deceased group compared with the surviving group (*P* < 0.01) (Fig. 6).

### Associations between lung and gut microbiota and 28-day mortality

Cox proportional hazards regression modeling was used to investigate potential associations between changes in gut and lung microbiota and 28-day mortality after mechanical ventilation. During the follow-up period, a total of 27 patients (44%) succumbed within 28 days. After an initial univariate analysis of variance, we subjected lung and gut microbial diversity, BALF *Enterobacteriaceae*, fecal *Enterococcaceae* and fecal *Enterococcus* to multivariate Cox regression analysis. Results showed that lung microbial diversity and fecal *Enterococcaceae* were correlated and independently associated with 28-day mortality (adjusted hazard ratio (aHR) = 0.773; 95% confidence interval (CI) 0.652 ~ 0.916, *p* = 0.003; aHR = 1.022; 95% CI, 1.008–1.037, *p* = 0.002) (Table 2). This result emphasizes the potential prognostic value of analyzing the gut and lung microbiota in predicting 28-day mortality in mechanically ventilated patients.

**Table 2:**
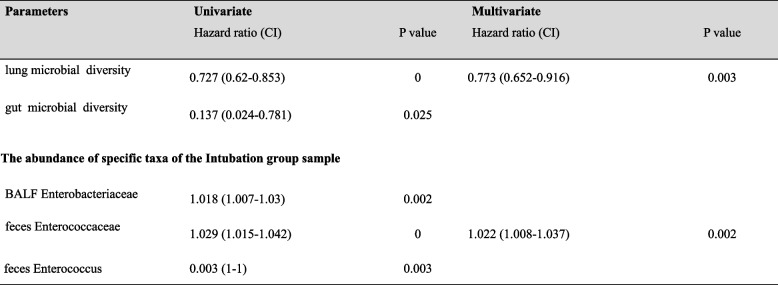
Cox regression analysis of risk factors associated with 28-day mortality in mechanically ventilated critically ill patients (*n* = 62)

Definition of abbreviations: *HR* hazard ratio, *aHR* adjusted hazard ratio, *95%CI*: 95% confidence interval

## Discussion

In this study, the taxonomic composition and diversity of the lung and gut microbiota of critically ill patients underwent profound changes. The results of this study reveal the predictive potential of lung microbiota dysbiosis and gut *Enterococcaceae* enrichment for 28-day mortality in mechanically ventilated critically ill patients. This study elucidates the gut-lung axis in terms of the impact of microbiota on the host and provides important insights into the adverse clinical outcomes associated with these changes.

A healthy intestinal microbiota is in a dynamic state of equilibrium, with each component mutually constraining the others [49]. Commensal bacteria can prevent the expansion of pathogens by mechanisms such as nutritional competition, indirect stimulation of host immunity, or support for epithelial barrier integrity [50, 51]. In previous studies, low fecal microbial diversity has been associated with an increased risk of death or infection, and the predominance of a single bacterial genus has been suggested to be an independent risk factor for poor outcomes [52, 53]. Our results confirm that lower microbial diversity in the lung is associated with a higher risk of mortality. The deceased lost important components of the intestinal commensal bacteria of healthy individuals, such as *Prevotellaceae*, *Veillonellaceae*, *Peptostreptococcaceae*, *Lachnospiraceae*, *Lactobacillaceae*, and others. Moreover, the concentration of Pentanoic acid, Butyric acid, Isobutyric acid, and Isovaleric acid in the feces of the deceased was decreased. Gut commensal bacteria produce SCFAs, which can modulate the immune response by influencing cell function through mechanisms such as participation in G protein-coupled receptor signaling (GPCR) and inhibition of histone deacetylase activity (HDAC) [54]. The gut commensal bacteria influence the body's defense mechanisms against lung infections [55–57]. Previous studies have shown that disruption of postnatal commensal colonization or selective loss of dendritic cells interrupts the IL-22 + ILC3 migratory program in the lung, rendering neonatal mice more susceptible to pneumonia. Conversely, postnatal translocation of commensal bacteria restores this ability and demonstrates that the presence of commensal bacteria is essential for neonatal mice to resist pneumonia [58, 59]. It is well-recognized that SCFAs have anti-inflammatory and immunomodulatory roles along the gut-lung axis [60]. Emerging evidence suggests that along the gut-lung axis, SCFAs can stimulate myeloid cells in the bone marrow, which then migrate to the lungs and form an anti-inflammatory environment [61]. Our results also showed a positive correlation between the concentration of SCFAs in the gut and clinical outcomes. The concentration of SCFAs could reflect the microbial ecological balance between the gut commensal microbiota and pathogens, and indirectly its protective effect against respiratory diseases, which could help to predict the clinical prognosis.

Studies have demonstrated a reduced diversity of lung and gut microbiota in mechanically ventilated patients [62, 63]. This phenomenon is independently associated with lower survival rates and longer duration of mechanical ventilation [64]. Similar to previous findings, our patients in the deceased group had reduced microbial diversity, with a significant reduction in microbial composition for gut commensals and an overgrowth of pathogenic dominant taxa (such as *Enterococcaceae* or *Enterobacteriaceae*) [65–67]. A single-center study involving 301 patients with rectal swabs taken at the time of ICU admission showed that Vancomycin Resistant Enterococci (VRE) colonization and *Enterococcaceae* predominant bacteria (30% and 15% of patients, respectively) were associated with mortality or all-cause infections [68]. Agreeable with our results, the enrichment of the gut microbiota in the lungs of critically ill patients is receiving increasing attention [69]. However, it is known that mechanical ventilation patients develop ARDS associated with lung microorganisms [70–72]. And yet, studies have also shown that the spread and transfer of commensal bacteria in the host's gut can lead to infectious complications in the lungs [73]. These findings are supported by the results of the present study, in which an enrichment of enteric bacteria, both commensal and pathogenic, was observed in the lungs of almost all patients in the ICU. The abundant presence of pathogenic enteric bacteria *Enterobacteriaceae* and *Enterococcaceae* in the lungs of patients in the deceased group was associated with their extensive colonization of the gut. It has been reported that it is possible for the microbiota to migrate to distant organs through the intestinal draining lymphatics, portal vein and systemic circulation when the intestinal barrier is compromised in critically ill patients. When intestinal and alveolar capillary permeability is increased, migration of the gut-lung microbiota is enhanced [74]. We propose that the enrichment of pathogenic bacteria of intestinal origin in the lungs parallels the gut microbiota. Gut flora dysbiosis leading to a microbial imbalance in the lungs, characterized by low microbial diversity and predominance of a single species may portend a poor outcome.

The human microbiota shows complexity, especially during critical illnesses when multiple factors can lead to microbiota dysbiosis. As it has been reported that aging is negatively correlated with the diversity of the gut microbiota [75]. Both the effects and mechanisms of antibiotic-driven gut microbiota disruption have been used as an important part of microbiology research in critically ill ICU patients [76, 77]. Although there was no difference in antibiotic use between the two groups in this study, the effect of antibiotics on the microbiome cannot be ruled out. Due to the common use of antibiotics in ICU patients, accompanied by the emergence of multidrug-resistant enteric bacteria, antibiotics may also bring unavoidable pathophysiological damage to produce a negative impact. Therefore, we emphasize microecological balance and avoid clinical operations that may interfere with microecological dysbiosis. For example, in the same way that antimicrobials often cause hepatic and renal impairment, microecological disturbances induced by antibiotics are emphasized and mentioned in routine clinical considerations [78]. In addition, probiotic supplementation, improvement of the intestinal environment through dietary interventions and synbiotics, or fecal microbiota transplantation (FMT) are common clinically active therapeutic measures. Probiotic supplementation has been applied to protect against the development of gut-bone marrow-lung axis associated lung injury. In mice fed a high-fiber diet with increased levels of circulating SCFAs, propionate increased the production of DC precursors in the bone marrow, causing lung resident DCs to be less effective in reactivating effector TH2 cells, thus avoiding allergic inflammation in the lungs [59]. Zhang demonstrates that propionic acid supplementation reverses Zinc Oxide Nanoparticle-Induced Lung Injury by acting on macrophages via receptor GPR43 [79]. Popular probiotic preparations such as Lactobacillus and Bifidobacterium strains generally exhibit beneficial properties. Intranasal administration of Lactobacillus in mice prevents hyperoxia-induced lung injury [80] and protects against Pseudomonas aeruginosa pneumonia in mice [81]. A sub-strain of Bifidobacterium, group BLBB, promotes an increase in short-chain fatty acid-producing strains [82]. Another sub-strain, Lactis BL-99, promotes up-regulation of acetate and butyrate receptors, including FFAR2, FFAR3 and GPR109a expression, which negatively correlates with monocytes and macrophages and ameliorates colitis-associated lung injury in mice [83]. Further studies are needed due to the multiple effects of microbial metabolites and the complexity of immune system mechanisms at the hematopoietic level. In the real world, Lactobacillus and Bifidobacterium demonstrate the same benefits. Probiotic supplementation formulated as Lactobacillus acidophilus LA-5, Lactobacillus plantarum, Bifidobacterium lactis BB-12, and Saccharomyces boulardii in mechanically ventilated polytraumatized patients intubated immediately after trauma reduces the incidence of VAP [84]. Administration of synbiotics (Bifidobacterium breve strain Yakult, Lactobacillus casei strain Shirota, and galactooligosaccharides) to mechanically ventilated septic patients was associated with elevated levels of acetate, with reduced incidence of enterocolitis and reduced incidence of VAP [85]. It has also been reported that probiotic supplementation in adult patients with covid-19 was well tolerated and reduced nasopharyngeal viral load, pulmonary infiltrates, and the duration of digestive and non-digestive symptoms [86]. Besides probiotics, the clinical role of FMT may be more significant, as complex microbial agents that act as a community, rather than as an isolated strain. It is observed that species with anti-inflammatory properties are increased in mice receiving FMT [87]. FMT and SCFAs restore a healthy proportion of gut microbes in mice and augment gut barrier proteins, reducing the release of inflammatory factors IL-1β and IL-18, which play a protective role in sepsi [88]. However, FMT is also used less often because of difficulties in implementation. Complications have also been reported with single probiotics [89]. Regarding the future of microbial therapy, not only safety and clinical convenience should be taken into account, but targeted complex probiotic preparations for patient-individualized flora may be more needed.

Although we do not have microbiological data for a longer period of time, it has been reported that there is a prolongation of microecological abnormalities in critically ill patients [16], even after discharge from the hospital [90]. Disturbed microecosystems may be the slowest part of the patient's overall recovery, and during this recovery process, the microecological flora is maintained in an abnormal steady state, which may be part of the organ sequels, increasing the risk of future exacerbations and admissions to the hospital [91]. Restoring microecological function as much as possible is part of our treatment and needs to be included in the assessment of efficacy. Food and lifestyle are the best choices we can make to restore our microbial environment and contribute to defense against disease, as well as improve disease prognosis.

Finally, it should be emphasized that this was a small, single-center study with the disadvantage that we could not guarantee a sufficient number of subjects in each group due to the clinical complexity of ICU patients and the use of multiple therapeutic measures. It was difficult for us to analyze important confounding factors such as age, gender, and severity of illness, which have a strong influence on human microbial variation. Therefore we cannot ignore this and the results of the multifactorial cox proportional risk regression cannot be fully generalized. In future studies, more participants need to be recruited to better stratify patients’ baseline characteristics and to collect more clinical data for etiologic studies.

## Conlusion

The lung and gut microbiota of critically ill patients undergo a marked shift upon admission to the ICU, which is further exacerbated over time. A significant decrease in lung microbial diversity and proliferation of *Enterococcaceae* in the gut were associated with 28-day mortality. The gut-lung axis opens up new possibilities for therapeutic approaches to respiratory diseases beyond the pulmonary environment. Understanding and modulating microbes to achieve therapeutic goals of preventing and treating critical illnesses should be included in the ICU physician's discussion of patient care. We recommend precise protocols tailored to the microbial characteristics of the patient and the addition of SCFAs-producing bacteria to provide intestinal and circulating levels of SCFAs for lung protection. In general, this study helps to improve clinicians' understanding of gut and lung microbiota dysbiosis and poor outcomes and provides direction for clinical microbiologic therapy.

## Data Availability

The raw sequence data reported in this paper have been deposited in the Genome Sequence Archive (Genomics, Proteomics & Bioinformatics 2021) at National Genomics Data Center (Nucleic Acids Res 2022), China National Center for Bioinformation / Beijing Institute of Genomics, Chinese Academy of Sciences (GSA: CRA010794) that are publicly accessible at (https://ngdc.cncb.ac.cn/gsa).

## Abbreviations

ICU: Intensive care
16S ribosomal RNA: DNA region coding for ribosomal 16S RNA subunit
SCFAs: Short-chain fatty acids
BALF: Bronchoalveolar lavage fluid
GCS: Glasgow Coma Scale
PEEP: Positive end-expiratory pressure
SOFA: Sequential Organ Failure Assessment
ARDS: Acute respiratory distress syndrome
SRA: Sequence Read Archive
OTUs: Operational taxonomic units
NMDS: Non-metric multidimensional scaling
PCoA: Principal coordinate analysis
VAP: Ventilator associated pneumonia
CRAB: Cabapemne Resistant Acinetobacter Baumannii
CRKP: Carbapenem-resistant Klebsiella pneumoniae
CRPA: Carbapenem-resistant Pseudomonas aeruginosa
MASA: Methicillin-susceptible Staphylococcus aureus
GPCR: G protein-coupled receptor signaling
HDAC: Histone deacetylase activity
FMT: Fecal microbiota transplantation
VRE: Vancomycin Resistant Enterococci
